# Oil accumulation mechanisms of the oleaginous microalga *Chlorella protothecoides* revealed through its genome, transcriptomes, and proteomes

**DOI:** 10.1186/1471-2164-15-582

**Published:** 2014-07-10

**Authors:** Chunfang Gao, Yun Wang, Yue Shen, Dong Yan, Xi He, Junbiao Dai, Qingyu Wu

**Affiliations:** MOE Key Laboratory of Bioinformatics, School of Life Sciences, Tsinghua University, Beijing, 100084 China; BGI-Shenzhen, Shenzhen, 518083 China; Department of Criminal Science and Technology, People’s Public Security University of China, Beijing, 100038 China

**Keywords:** Microalgae, *Chlorella protothecoides*, Genome sequence, Proteomic, Oil accumulation, Hexose-proton symporter, Transcriptome, Lipid

## Abstract

**Background:**

Microalgae-derived biodiesel is a promising substitute for conventional fossil fuels. In particular, the green alga *Chlorella protothecoides* sp. 0710 is regarded as one of the best candidates for commercial manufacture of microalgae-derived biofuel. This is due not only to its ability to live autotrophically through photosynthesis, but also to its capacity to produce a large amount of biomass and lipid through fermentation of glucose. However, until the present study, neither its genome sequence nor the platform required for molecular manipulations were available.

**Results:**

We generated a draft genome for *C. protothecoides*, and compared its genome size and gene content with that of *Chlorella variabilis* NC64A and *Coccomyxa subellipsoidea* C-169. This comparison revealed that *C. protothecoides* has a reduced genome size of 22.9 Mbp, about half that of its close relatives. The *C. protothecoides* genome encodes a smaller number of genes, fewer multi-copy genes, fewer unique genes, and fewer genome rearrangements compared with its close relatives. In addition, three *Chlorella*-specific hexose-proton symporter (HUP)-like genes were identified that enable the consumption of glucose and, consequently, heterotrophic growth. Furthermore, through comparative transcriptomic and proteomic studies*,* we generated a global perspective regarding the changes in metabolic pathways under autotrophic and heterotrophic growth conditions. Under heterotrophic conditions, enzymes involved in photosynthesis and CO_2_ fixation were almost completely degraded, either as mRNAs or as proteins. Meanwhile, the cells were not only capable of quickly assimilating glucose but also showed accelerated glucose catabolism through the upregulation of glycolysis and the tricarboxylic acid (TCA) cycle. Moreover, the rapid synthesis of pyruvate, upregulation of most enzymes involved in fatty acid synthesis, and downregulation of enzymes involved in fatty acid degradation favor the synthesis of fatty acids within the cell.

**Conclusions:**

Despite similarities to other *Chlorella*, *C. protothecoides* has a smaller genome than its close relatives. Genes involved in glucose utilization were identified, and these genes explained its ability to grow heterotrophically. Transcriptomic and proteomic results provided insight into its extraordinary ability to accumulate large amounts of lipid. The *C. protothecoides* draft genome will promote the use of this species as a research model.

**Electronic supplementary material:**

The online version of this article (doi:10.1186/1471-2164-15-582) contains supplementary material, which is available to authorized users.

## Background

World demand for fuel, currently supplied mainly in the form of non-renewable fossil fuel, is continuously rising, leading to an increased interest in alternative and sustainable energy sources. Biodiesel derived from microalgae has opened up a new, promising path to solve the energy crisis [1]. Compared with oil-producing plants, the most attractive advantage of microalgae is their high oil production capacity; the annual oil yield per hectare is 10 times higher than that of oil seed crops [2]. However, to meet industrial scale requirements, the cost of biofuel production must be further reduced [2]. One promising strategy is to apply technologies developed in metabolic engineering and synthetic biology. However, this approach requires an oleaginous algal model with high oil production capacity, known genetic information, and established methods for molecular manipulation.

*Chlorella protothecoides* sp. 0710 (hereafter referred to as Cp0710) is a unicellular green alga with great potential for biodiesel production. It has been proposed as one of the best candidates for commercial manufacture of microalgae-derived biofuel [3]. One special characteristic that distinguishes Cp0710 from many other algae is its ability to not only live autotrophically through photosynthesis but also to grow heterotrophically by using extracellular organic carbon sources such as glucose [3–5]. Furthermore, when the organism switches from autotrophism to heterotrophism, the chloroplast disappears and is replaced by lipid bodies, leading to high oil accumulation [6]. Based on this finding, a photosynthesis fermentation approach (PFA) was developed to cultivate Cp0710. This approach combines autotrophic and heterotrophic growth, resulting in 69% higher oil yield and 61.5% less CO_2_ emission compared with typical heterotrophic cultivation [7]. In addition, alternative carbon sources and the combination of different cultivation approaches have been applied to further reduce the cost and to boost biomass/lipid production [8–11]. Despite all of this progress in recent years, both the genome sequence of Cp0710 and the platform for molecular manipulations are still missing.

In the present study, we generated the whole-genome sequence of Cp0710 using multiple strategies, and built a *de novo* assembly, which demonstrated that this alga has a small genome that encodes the lowest number of genes among all sequenced green microalgae. The Cp0710 genome was annotated and characterized by comparative genomic analysis. In addition, to investigate the differences between autotrophic and heterotrophic cells in metabolic pathways and related regulation patterns, differential expression of mRNA and proteins in the two cell types were studied and a potential regulatory pathway that could result in accumulation of oil in heterotrophically growing Cp0710 was identified. Together, the genome, transcriptome, and proteome analyses presented in this study yield new insights into the molecular basis for the accumulation of oil and provide a rich resource of genetic information that will be useful for the development of Cp0710 as an oleaginous model microalga.

## Results

### Genome sequencing, assembly, and annotation

A whole-genome shotgun strategy was adapted to sequence the genome of Cp0710 using both Roche and Illumina technologies. A total of 4.3 Gbp of clean data, representing 159-fold coverage of the estimated genome was used for assembly, producing a genome assembly with scaffold L50 of 285,534 nucleotides (nts) and contig L50 of 35,091 nts (Table 1). The size of the assembled Cp0710 genome was estimated to be 22.9 Mbp. Pulse field gel electrophoresis revealed at least six discrete bands ranging from 800 Kbp to 2,000 Kbp (Additional file 1 Figure S1), suggesting at least six different-sized chromosomes.

**Table 1:** **The contigs and scaffolds of** ***C. protothecoides*** **sp. 0710 genome**

	Contig		Scaffold	
	Size (bp)	Number	Size (bp)	Number
N90	9361	654	87711	74
N50	35091	195	285534	24
Longest	150212	-	1144347	-
Total_Size	21873284	-	22929133	-
Number (>100 bp)	-	1419	-	406
Number (>2 kb)	-	1017	-	187

To assess genome coverage and completeness of the whole genome, we also estimated the genome size for Cp0710 utilizing 17-base k-mers and their Poisson distribution in the sequencing reads [12]. The estimated Cp0710 genome size was 27.6 Mbp (Additional file 1 Figure S2 and Additional file 1 Table S1). Considering the presence of sequencing errors, the sequencing depth is expected to be underestimated and, consequently, the Cp0710 genome size should be smaller than 27.6 Mbp. Using this estimated genome size, the assembled contigs and scaffolds should at least cover about 80% and 83% of the whole genome, respectively. Further, the completeness of the genome was assessed using CEGMA v2.4 (Core Eukaryotic Genes Mapping Approach) based on mapping of the 248 most highly conserved core eukaryotic genes (CEGs) [13]. The completeness of the assembled genome was 90.73% (Additional file 1 Table S2).

Overall, the nuclear genome was 63% G/C (Table 2), which is similar to *Chlamydomonas reinhardtii* (64%) but slightly lower than *Chlorella variabilis* NC64A (67%) and higher than *Coccomyxa subellipsoidea* C-169 (53%). The Cp0710 genomic sequence was repeat poor, with only about 6.1% of the genome composed of repetitive sequences, making it the Trebouxiophyceae genome with the fewest repeats (Table 2 and Additional file 1 Table S3). Known transposon-derived repetitive sequences accounted for less than 1.1% of the genome, with LTR retrotransposons, non-LTR retrotransposons, and DNA transposons making up 0.29%, 0.39%, and 0.42% of the genome, respectively (Additional file 1 Table S4).

**Table 2:** **Genomic features of sequenced chlorophyte green algae**

	CPRO	CVAR	CSUB	CREI	VCAR	MPUS	OTAR
Taxonomic class	T	T	T	C	C	M	M
Assembly length (Mb)	22.9	46.2	48.8	121	138	21.9	12.6
GC content (%)	63	67	53	64	56	65	58
Repeat sequences (%)	6.1	8.9	7.2	16.7	23.8	8.8	5.1
Number of gene	7,039	9,791	9,851	15,143	14,520	10,575	8,166
Average gene length (bp)	2,863	2,928	3,503	4,312	5,269	1,557	nd
Average number of exons per gene	5.72	7.3	8.2	8.33	7.78	1.9	1.57
Average exon length (bp)	207	170	182	190	194	731	750
Mean length of introns (bp)	246	209	240	373	491	187	103
Coding sequence ratio (%)	3.2	4.7	5.0	8.0	9.5	2.1	1.6

*CPRO*: *Chlorella protothecoides* sp.0710; *CVAR*: *Chlorella variabilis* NC64A; *CSUB*: *Coccomyxa subellipsoidea* C-169; *CREI*: *Chlamydomonas reinhardtii*; *VCAR*: *Volvox carteri*; *MPUS*: *Micromonas pusilla* CCMP1545; *OTAR*: *Ostreococcus tauri*.

*T*: Trebouxiophyceae; *C*: Chlorophyceae; *M*: Mamiellophyceae.

**Figure 1:**
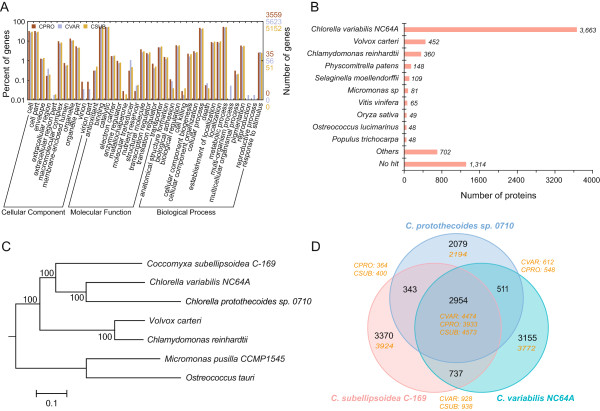
***Chlorella protothecoides*** **sp. 0710 is closely related to** ***Chlorella variabilis*** **NC64A. A)**. Genes in Cp0710, *C. variabilis* NC64A, and *C. subellipsoidea* C-169 were annotated by GO. Each bar represents the number of genes, and the different species are color-coded. **B)** The proteins in Cp0710 were searched by BLASTP against the TrEMBL protein database. The number of best-hit proteins in each organism is presented. **C)** A maximum likelihood phylogenetic tree was constructed using 300 single-copy orthologous genes shared in the seven species. **D)** The gene families in the three sequenced Trebouxiophyceae strains were compared. The Venn diagram shows the shared and unique gene families among the species. The number of gene families is indicated in black and the total number of genes is in yellow. CPRO: *C. protothecoides*, CVAR: *C. variabilis* NC64A; CSUB: *C. subellipsoidea* C-169.

A number of methods were used to predict genes, including homology-based methods and *de novo* gene prediction (Additional file 1 Table S5). These results were then integrated to generate a consensus gene set. In addition, the transcription information from RNA-seq was incorporated, resulting in a total of 7,039 genes in the genome. Of the 7,039 predicted genes, 6,800 (96.6%) were transcribed in either autotrophic or heterotrophic growing cells and 5,831 (82.9%) genes could be functionally annotated by homology search against multiple protein databases (Additional file 1 Table S6), including TrEMBL, Swissprot, KEGG, InterPro, and Gene Ontology (GO). The 3,559 genes annotated by GO were classified as encoding cellular components, proteins with molecular functions, or proteins involved in biological processes. The most abundant genes in the “molecular function” category were binding and catalytic genes. In the “biological processes” category, genes involved in cellular and metabolic processes were most abundant. No obvious enrichment or depletion of genes in specific GO groups was found in comparison with *C. variabilis* NC64A and *C. subellipsoidea* C-169 (Figure 1A and Additional file 1 Figure S3).

In addition, the complete sequences of the mitochondrial and chloroplast genomes were recovered from sequencing reads after gap-repair, revealing two circular molecules that were 57,274 bp and 84,580 bp in size, respectively. The detailed analysis of these genomes will be reported elsewhere (Yan et al., manuscript in preparation).

### Genome characterization

#### Comparative genomics

*C. protothecoides* is a member of the class Trebouxiophyceae in the phylum Chlorophyta. Presently, only two strains in the Trebouxiophyceae, *C. variabilis* NC64A [14] and *C. subellipsoidea* C-169 [15], have been fully sequenced. To gain insight into the novel features of the Cp0710 genome, we compared it with not only these two strains, but also several other sequenced chlorophytes (Table 2), including two in the Chlorophyceae (*C. reinhardtii* and *Volvox carteri)* and two in the Mamiellophyceae (*Micromonas pusilla* CCMP1545 and *Ostreococcus tauri)*.

The size of the genome was regarded as a consequence of evolution [16], as evolutionarily related species usually have similar genome size. Accordingly, *M. pusilla* CCMP1545 and *O. tauri*, two ancient green algae belonging to the Prasinophyceae [17, 18], have the smallest genomes, at 22 Mbp and 13 Mbp, respectively. In fact, of the available algal genome sequences, all members of the Prasinophyceae contain genomes that range in size from 12 to 22 Mbp [19]. On the other hand, among the five sequenced chlorophytes, genome sizes range from 46 Mbp (*C. vulgaris* NC64A) to as large as 140 Mbp (*V. carteri*), and the model green alga *C. reinhardtii* has a 121 Mbp genome. These values are consistent with the hypothesis that differences in genome size correlate with the evolutionary position of related species. Surprisingly, though, the size of the Cp0710 genome is only 22.9 Mbp, making it the smallest genome reported so far in the sequenced chlorophytes, and falling in the range of the prasinophytes. *C. variabilis* NC64A and *C. subellipsoidea* C-169, the two closest relatives, have genomes that are double this size, suggesting that a dramatic change in the size of the genome happened after the diversification of these algae.

The unexpectedly small size of the Cp0710 genome led us to reconsider its origin and its evolutionary relationship with other *Chlorella* species. Therefore, we analyzed its coding proteins by comparing them with the TrEMBL protein database. The total number of best-hit proteins from all organisms was counted and is shown in Figure 1B. The result indicated that the majority of proteins encoded in the Cp0710 genome possess best-hit homologs in *C. variabilis* NC64A, and these account for 64% of the total proteins. Only 452 (6.4%) and 360 (5.1%) proteins had best-hit homologs in *V. carteri* and *C. reinhardtii*, respectively. In addition, although the average G/C content of Cp0710 was slightly lower than that of *C. variabilis* NC64, both genomes had G/C content greater than 60% (Table 2), consistent with the high G/C feature in the genomes of *Chlorella*. Furthermore, using 300 single-copy orthologues among the seven green algae, we built a maximum likelihood phylogenetic tree that indicated that *C. variabilis* NC64A is the closest relative of Cp0710 (Figure 1C). Finally, we compared the gene families among the three closest species (*C. protothecoides*, *C. variabilis* NC64A, and *C. subellipsoidea* C-169). As shown in Figure 1D, of the 5,887 gene families in Cp0710, nearly 50% (2954) are shared with *C. variabilis* NC64A and *C. subellipsoidea* C-169. There are 3,465 shared gene families between Cp0710 and *C. variabilis* NC64A, which account for 58.9% and 47.1% of the total gene families, respectively. Further comparison with the model alga *C. reinhardtii* identified a large number of unique genes (10,599; ~64%) in this species, suggesting a more distant relationship between *C. reinhardtii* and the other three green algae (Additional file 1 Figure S4). Together, these results confirmed the evolutionary proximity of Cp0710 and *C. variabilis* NC64A.

**Figure 2:**
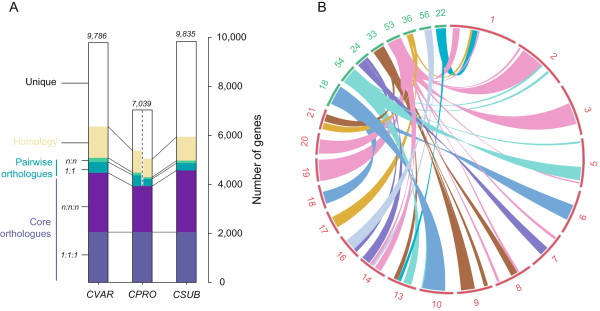
**Cp0710 has a compact genome. (A)** Comparison of genes in three sequenced Trebouxiophyceae. Genes are classified as core orthologues, pairwise orthologues, or homologous and unique genes, and were marked with different colors. The height of the bar represents the number of genes in each category. **(B)** Synteny comparison between Cp0710 and *C. variabilis* NC64A. The conserved blocks of synteny are indicated by a different color. Scaffold ID of Cp0710 and *C. variabilis* NC64A are indicated in green and red, respectively. Only scaffolds with a length greater than 1 Mbp and 400 Kbp in Cp0710 and *C. variabilis* NC64A, respectively, and the lengths of syntenic regions greater than 50 Kbp are shown.

Given that Cp0710 is a member of *Chlorella*, we then asked what kind of genomic features could have led to the drastically reduced genome size. As a first possibility, repetitive sequences are usually a big contributor to genome size, and this is especially true in plants [20]. Repeat analysis indicated that Cp0710 did, in fact, contain very few repetitive sequences, which altogether only accounted for 6.1% of the whole genome (Table 2). However, comparing this percentage with *C. variabilis* NC64A and *C. subellipsoidea* C-169, in which repeated sequences represent 8.9% and 7.2% of the genome, respectively, the reduced number of repetitive sequences would not be expected to result in the over 50% reduction in genome size between these species. Therefore, we proceeded to analyze the number of protein-coding genes in the three algae. As mentioned above, the total number of predicted genes in Cp0710 was only 7,039, which is substantially less than the nearly 10,000 genes predicted in the other two species. Further, we compared the genes among the three algae and clustered each of them into four categories (Figure 2A): core orthologues (including 1:1:1 orthologues and n:n:n orthologues), pairwise orthologues, homologs, and unique genes. We found that core orthologues, pairwise orthologues, and homologous genes were only slightly decreased in Cp0710; however, the number of genes in the n:n:n orthologues were greatly reduced (1,879 vs. 2,420 and 2,519 in *C. variabilis* NC64A and *C. subellipsoidea* C-169, respectively). Since the n:n:n orthologues represent the number of duplicated genes in the organism, this result indicated that the core set of genes within the three algae is similar but in the Cp0710 genome, the copy number of each gene is reduced. Meanwhile, the number of unique genes also differed greatly between Cp0710 and the others. There were only 2,194 genes present in the Cp0710 genome, compared with 4,700 and 4,862 unique genes in the other two species. This could presumably result in a significant decrease in genome size. Furthermore, we performed a whole-genome alignment of Cp0710 and *C. variabilis* NC64A. The aligned gapless segments with the same order and orientation in both species were ligated into a “chain” to generate the synteny map shown in Figure 2B. This analysis demonstrated that multiple copy syntenic regions were present in the *C. variabilis* NC64A genome. For example, four large regions (segments 2, 3,19, and 20) and several small regions (in pink) in the *C. variabilis* genome were similar to segment 53 in Cp0710, suggesting genome duplication/rearrangement happened at this location. Therefore, through the different analyses described above, it can be concluded that the reduced gene numbers (both the total number of genes and the redundant copies), including fewer unique genes and the lack of the genome duplication/rearrangement seen in *C. variabilis*, may, collectively, contribute to the smaller genome size of Cp0710.

### Nitrogen transport and assimilation

In plants, both carbon (C) and nitrogen (N) are crucial for many fundamental cellular activities, and are required not only as nutrients but also as environmental signals to control metabolism and cellular responses [21]. Different organisms have their own preferred N sources, and there are different transporters and assimilation systems involved. For Cp0710, we showed in an earlier study that it prefers ammonium and amino acids as nitrogen sources, and it has lost the ability to grow on nitrate- or urea-containing medium. We also identified and characterized one ammonium transporter gene (*CpAMT1*) [22]. To further our understanding of the nitrogen preference of Cp0710, a survey of proteins involved in nitrogen transport and assimilation in the Cp0710 genome was performed, leading to the identification of 13 genes (Additional file 1 Table S7). Not surprisingly, both the nitrate and the urea transporter were missing from the Cp0710 genome but were present in other green algae. Meanwhile, three nitrogen-related transporters (the nitrite, ammonium, and amino acid transporters) were identified, consistent with our findings on the choice of nitrogen sources in Cp0710, as described previously [22].

The ornithine-urea cycle (OUC), a metabolic pathway that is important for detoxification of excessive ammonia in animals [23], is absent from green plants and algae, though it was recently identified in diatoms [24]. Similarly, the Cp0710 genome does not encode the requisite proteins for a complete OUC; it has lost two of the key enzymes: arginase, which catalyzes the conversion of arginine to ornithine and urea, and urease, which breaks down urea to produce ammonia. However, Cp0710 has all the genes necessary for the aspartate-argininosuccinate shunt, which connects the OUC and tricarboxylic acid (TCA) cycles and is well-established in animal cells [23]. Recently, a similar connection was also found in diatoms, which included not only OUC and TCA cycles, but also the glutamine synthetase/glutamate synthase cycle, although the biological function of this coupling in these photosynthetic microalgae are still not clear [24]. The presence of these genes suggested a conserved pathway might also exist in Cp0710. To further support this hypothesis, we also identified the nitrogen regulatory protein PII and glutamate synthase, two key components in regulating carbon and nitrogen metabolism [25]. Nitrogen regulatory protein PII is a sensor of 2-oxoglutarate and ATP, while glutamate synthase catalyzes glutamate synthesis from 2-oxoglutarate. Both of these link the TCA cycle and nitrogen assimilation through 2-oxoglutarate, and thus control the flow of carbon and nitrogen.

The linkage between carbon and nitrogen metabolism might be especially important in Cp0710, since the interchange between autotrophic and heterotrophic growth is achieved through the availability of C and N sources (or C/N ratio). High nitrogen supplementation in the media will block the ability of the cells to switch from autotrophic to heterotrophic growth, as indicated by persistent chlorophyll, regardless of the presence or absence of organic carbon sources. Future study will focus on understanding how the carbon and nitrogen signal is detected and transferred, and whether and/or how this signal will directly contribute to the high oil content in Cp0710.

### Carbohydrate transporters

To utilize glucose from the heterotrophic medium, Cp0710 must be able to actively transport the sugar into cells. This task is normally carried out by H^+^/hexose co-transporters [26–28]. In *Chlorella kessleri*, a species which is also capable of using glucose to support cell growth, three H^+^/hexose co-transporter genes (*HUP1*, *HUP2*, and *HUP3*) have been identified [29]. Therefore, we performed homologous search using the HUPs from *C. kessleri* as queries, and identified homologs in all seven green algae species (Additional file 1 Table S8). However, the number of homologous proteins among these algae was different. They were most abundant in *C. subellipsoidea* C-169 (12 proteins), and 9 proteins were identified in Cp0710.

To evaluate the evolutionary relationship of the HUPs, a phylogenetic tree was generated (Additional file 1 Figure S5). The homologs of H^+^/hexose co-transporters were categorized into three classes, of which two were common in green algae. Interestingly, the third class, which includes the three HUP proteins, was only present in the three *Chlorella* species and the other related alga *C. subellipsoidea* C-169. This result illustrated that these HUPs were restricted to *Chlorella*, suggesting that the inability of other green algae to utilize glucose might be due to the absence of these proteins. In addition, hydrophobicity analysis of the three HUP-like proteins (Cpr004256.1, Cpr001753.1, and Cpr003452.1) indicated that each of them contained 12 transmembrane domains, similar to the prototypical HUP proteins in *C. kessleri* [30]. Moreover, it has been shown that six conserved amino acid residues are responsible for hexose recognition [31], and we found that all of them are conserved in the HUP-like proteins in Cp0710 (Additional file 1 Figure S6). Therefore, our data suggested that the three identified HUP-like proteins might be responsible for the ability of Cp0710 to rapidly utilize glucose under heterotrophic conditions. Studies on these glucose transporters are being actively pursued.

### Transcriptomes and proteomes under autotrophic and heterotrophic growth conditions

To dissect the potential mechanisms underlying the highly elevated oil accumulation in heterotrophic cells, the transcriptomes and proteomes of cells under the two growth conditions were analyzed. A total of 40.2 M and 37.9 M clean reads were obtained from RNA-seq in autotrophic and heterotrophic samples, with 80.7% and 81.2% mapping ratios to the genome, respectively (Additional file 1 Table S9). Overall, 30.4% of the genes were expressed differently during heterotrophic growth, with 984 over-expressed and 1,136 downregulated (Additional file 2 Table S10). In addition, through one dimension protein separation followed by mass spectrometry analysis, we identified a total of 1,931 proteins (Additional file 1 Figure S7). Among these, 674 proteins had peptide spectrum matching (PSM) ≥ 5, and these were used for the subsequent differential expression analysis. Compared with the autotrophic cells, in heterotrophic cells, 205 proteins were upregulated while 293 proteins were downregulated. In addition, comparison between the proteomic and transcriptomic data indicated that the two datasets largely agreed with each other (Additional file 3 Figure S11), despite the fact that fewer genes were identified in the proteomic study, demonstrating the reliability of both methodologies.

**Figure 3:**
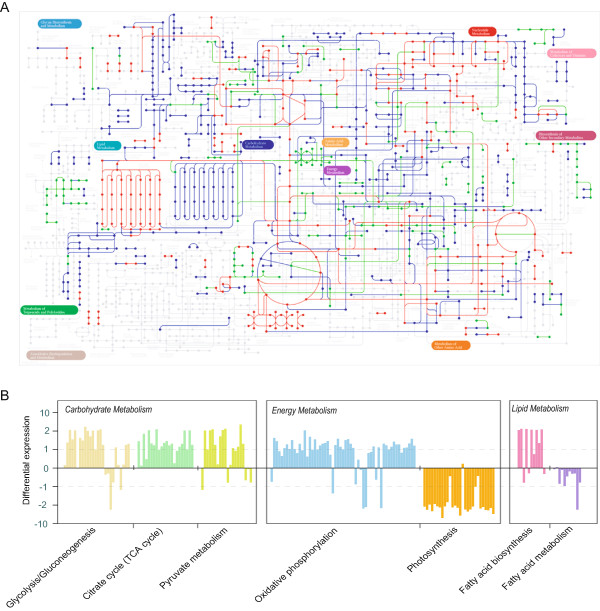
**Multiple metabolic pathways are differentially regulated under heterotrophic conditions. (A)** The whole KEGG pathway map after integrating the RNA-seq data. The gray lines indicate pathways not present in Cp0710 and the blue lines represent pathways that showed no significant changes in transcription. Pathways enriched in genes that are upregulated or downregulated are labeled in red and green, respectively. Significance is defined as log2 (fold-change) >1 and P < 0.01. Transcripts for enzymes with multiple subunits or isoforms were summed. **(B)** Selected pathways that are uniformly changed in heterotrophic cells. Only carbohydrate metabolism, energy metabolism, and lipid metabolism are listed here. See Additional file 1 Figure S9 for a complete list of affected pathways.

Next, we performed functional annotation and pathway analysis of the 2,120 differentially expressed genes using KEGG pathway annotation (Figure 3A and Additional file 3 Table S11). Red and green lines indicate that the genes involved in the pathways are either upregulated or downregulated, respectively. Blue lines represent pathways with no significant changes between the two types of cells. As shown in Figure 3A, this analysis identified several pathways that were enriched for either upregulated or downregulated genes. Zooming in on the pathways, we found that all of the differentially expressed genes could be classified into one of nine clusters (Additional file 1 Figure S9), and the most affected processes were the ones involved in carbohydrate, energy, and lipid metabolism as shown in Figure 3B. In addition, similar analysis was performed based on GO annotation, which revealed a similar pattern (Additional file 4 Table S12).

Compared with autotrophic cells, the strongly upregulated genes in heterotrophic cells are involved in glycolysis/gluconeogenesis, the TCA cycle, pyruvate metabolism, oxidative phosphorylation, and fatty acid biosynthesis (Figure 3B, Additional file 1 Figure S9 and Additional file 4 Table S12). This result is not unexpected, since the increase in glycolysis, pyruvate metabolism, and the TCA cycle could result in the rapid accumulation of ATP and other important precursors such as acetyl-CoA, which are critical for fatty acid synthesis in heterotrophic algae. In addition, the pentose phosphate pathway is another significantly upregulated process. This pathway is important for glucose consumption coupled with NADPH generation, and the latter is necessary for fatty acid synthesis. Furthermore, energy metabolism shifts dramatically from photophosphorylation to oxidative phosphorylation under heterotrophic conditions, suggesting a high demand for energy, presumably for rapid cell growth and lipid accumulation. Overall, all of the upregulated pathways can directly or indirectly enhance the accumulation of fatty acids in heterotrophic algae.

**Figure 4:**
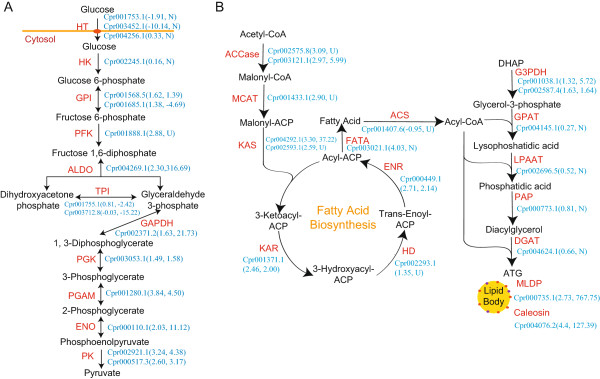
**Glycolysis and the triacylglycerol biosynthetic pathway in Cp0710. (A)** Change of enzyme expression in glycolysis under heterotrophic growth conditions. The name of each enzyme is in red and the corresponding protein ID in Cp0710 is shown in blue. Values in brackets indicate changes in RNA (left) and protein (right) levels, respectively. U: only identified in heterotrophic approach (upregulation). D: only identified in autotrophic approach (downregulation). N: not detected. **(B)** Change in enzyme expression in the triacylglycerol biosynthetic pathway under heterotrophic growth conditions. The color code is the same as in **(A)**. Abbreviations of enzymes are as follows: HT, H^+^/hexose co-transporter; HK, hexokinase; GPI, glucose-6-phosphate isomerase; PFK, 6-phosphofructokinase; ALDO, fructose-bisphosphate aldolase; TPI, triose phosphate isomerase; GAPDH, glyceraldehyde 3-phosphate dehydrogenase; PGK, phosphoglycerate kinase; PGAM, phosphoglycerate mutase; ENO, enolase; PK, pyruvate kinase; Accase, acetyl-CoA carboxylase; MCAT, acyl-carrier protein (ACP) S-malonyltransferase; KAS, 3-oxoacyl-ACP synthase II; KAR, 3-oxoacyl-ACP reductase; HD, 3-hydroxyacyl-ACP dehydratase; ENR, enoyl-ACP reductase I; FATA, fatty acyl-ACP thioesterase A; ACS, long-chain acyl-CoA synthetase; G3PDH, glycerol-3-phosphate dehydrogenase; GPAT, glycerol-3-phosphate O-acyltransferase 3/4; LPAAT, lysophosphatidate acyltransferase; PAP, phosphatidate phosphatase; DGAT, diacylglycerol O-acyltransferase 1; MLDP, major lipid droplet protein.

On the other hand, there were a few pathways that were dramatically downregulated. These pathways included photosynthesis, porphyrin and chlorophyll metabolism, and carotenoid biosynthesis (Figure 3B, Additional file 1 Figure S8, and Additional file 4 Table S12). Downregulation of photosynthesis under heterotrophic growth conditions is to be expected, since the availability of glucose in the medium will allow the algae to stop using photosynthesis to obtain organic carbon. More importantly, the elimination of chloroplasts, which usually occupy a large volume in autotrophic cells, will presumably provide a lot of space for the accumulation of lipid bodies in heterotrophic cells (Additional file 2 Figure S10). In addition, genes involved in fatty acid degradation are uniformly downregulated (Figure 3B), which presumably may allow the cells to keep the fatty acids in the form of triacylglycerol as an energy reserve, making this alga optimized for lipid production.Furthermore, we integrated the proteomic and transcriptomic data into the metabolic pathways for glucose utilization and fatty acid and triacylglycerol biosynthesis under heterotrophic growth conditions. As shown in Figure 4A and 4B, the proteomic data largely agree with the transcriptomic results, in which a majority of the enzymes in these pathways are highly expressed. For some key enzymes, such as fructose-1, 6-bisphosphate aldolase (ALDO), which catalyzes the conversion of fructose-1, 6-bisphosphate to glyceraldehyde 3-phosphate and dihydroxyacetone phosphate, the protein level increased over 300 times. In addition, both RNA and protein levels of the major lipid droplet protein (MLDP) were boosted, consistent with the accumulation of lipid droplets in heterotrophic cells.

## Discussion

Cp0710 is a green alga capable of growing both autotrophically and heterotrophically, and accumulating large amounts of lipid within the cells under heterotrophic growth conditions. This phenotype implies that a specific mechanism, though still elusive, may be encoded in its genome. Here, we generated the first draft of the genome sequence of Cp0710, and also analyzed the transcriptomes and proteomes for cells growing under autotrophic and heterotrophic conditions. One of the biggest surprises resulting from our analyses was that Cp0710 contains a very small nuclear genome, about half the size of the *C. variabilis* NC64A or *C. subellipsoidea* C-169 genome. The high degree of similarity of the proteins of Cp0710 and *C. variabilis* NC64A indicates that the two algae are evolutionarily related, and that Cp0710 is a *bona fide* species of *Chlorella*. Meanwhile, several possible reasons for the smaller genome in Cp0710 were considered, including fewer encoded genes, fewer copies of orthologues, and fewer genome duplications and rearrangements (Figure 2 and Table 2).

Another interesting finding after analyzing the Cp0710 genome was the lack of both nitrate and urea transporters, while other nitrogen-related transporters were present, including nitrite, ammonium, and amino acid transporters. This result was not a surprise to us since our previous study showed that this alga could not grow in medium using nitrate or urea as nitrogen sources [22]; however, no other green alga sequenced so far has lost both of these transporters. Since *C. variabilis* NC64A and *C. subellipsoidea* C-169 have both transporters, it is possible that the loss of these transporters from the Cp0710 genome might have happened recently, after the divergence of these species from their last common ancestor. On the other hand, the increased number of amino acid transporters, as revealed by domain analysis using PFAM (Additional file 3 Figure S11), suggests that Cp0710 has adapted to an environment rich in amino acids, and this is consistent with its previously reported N-source preference [32].

Our study unveiled a fine-tuned pathway for lipid synthesis in Cp0710 under heterotrophic growth conditions. First, the glucose in the medium is transported into the cell via the glucose transporters Cpr004256.1, Cpr001753.1, and Cpr003452.1. Preliminary data also suggested that the three transporters responded differently upon exposure to media containing different amounts of glucose. This was first hinted at by the transcriptomic comparison between autotrophic and heterotrophic cells. One or more of these transporters may enable the alga not only to grow heterotrophically, but also to accumulate large amounts of glucose in the cell to fulfill the energy requirement for lipid synthesis. Then, after glucose enters the cells, it is broken down through glycolysis and the TCA cycle. In heterotrophic cells of Cp0710, nearly all the glycolytic enzymes, including rate-limiting enzymes such as 6-phosphofructokinase (Cpr001888.1) and pyruvate kinase (Cpr002921.1 and Cpr000517.3) were significantly increased (Figure 4A). The TCA cycle was also enhanced under heterotrophic conditions. The increased production of the enzymes in these two pathways ensured a high rate of catabolism of glucose to meet the demand for energy and intermediates for rapid growth and lipid synthesis. As such, high glycolytic ability may be one of the bases for the ability of Cp0710 to accumulate large amounts of lipid. By contrast, a similar function in *C. reinhardtii* seems much weakened despite the fact that this species can also utilize glucose after a transporter is introduced. However, its growth under heterotrophic conditions is limited and the cells fail to accumulate large amounts of lipids [33]. Secondly, acetyl-coenzyme A carboxylase (ACCase) is regarded as one of the key enzymes in regulating the carbon flux to fatty acid biosynthesis, and over-expression of this enzyme could increase the lipid content [34]. Coincidentally, we found that expression of both the biotin carboxyl carrier subunit (Cpr002575.8) and the biotin carboxylase (Cpr003121.1) subunit of the heteromeric ACCase were elevated in heterotrophic cells (Figure 4A). In addition, all components of the fatty acid synthase subunits, which catalyze the elongation of fatty acids, are also upregulated (Figure 4B). The presence and elevated expression of these enzymes indicated that Cp0710 has adopted a unique pathway to ensure the rapid biosynthesis of fatty acids. Finally, the fatty acyl-CoA generated during *de novo* synthesis is esterified to make TAG, which will eventually be stored inside the lipid bodies. This requires glycerol-3-phosphate (G3P), a product derived from either glycerol or dihydroxyacetone phosphate (DHAP). In Cp0710, the upregulation of the two isozymes of glycerol-3-phosphate dehydrogenase (Cpr001038.1 and Cpr002587.4), which produce G3P from DHAP, ensures the supply of G3P. To meet the cells’ consequent requirement for DHAP, the alga adopted a mechanism generally used in higher plants: inactivation of triose phosphate isomerase (encoded by Cpr001755.1 and Cpr003712.8 in Cp0710, Figure4B) [35, 36], a key enzyme that catalyzes the isomerization between DHAP and glyceraldehyde 3-phosphate (GAP), directly linking glycolysis and TAG synthesis. A previous study revealed that deficiency of triose phosphate isomerase increased the fatty acid or oil content in root cells of plants [35]. Therefore, we speculate that in Cp0710, a similar mechanism is deployed and the triose phosphate isomerase may be the key enzyme in regulating the carbon flux to fatty acid synthesis.

## Conclusion

We report in this paper an annotated draft genome of the oleaginous alga Cp0710, a candidate for use as a model alga for biofuel production. Several unique features of the genome were revealed in this analysis. First, the genome size is greatly reduced and the gene number is relatively small, but most of the genes are homologous to *C. variabilis* NC64A, implying that Cp0710 is evolutionarily related but probably distant from other *Chlorella*. Secondly, the undersized genome of Cp0710 may be due to a smaller number of genes encoded in the genome, fewer copies of duplicated and unique genes, and/or a more ancient genome with fewer duplications and rearrangements. Thirdly, three HUP-like genes, which are unique to *Chlorella*, were identified in the genome, providing a molecular basis for Cp0710 to be able to consume glucose and grow heterotrophically. Finally, Cp0710 is the only sequenced green alga that lacks both the nitrate and urea transporter, suggesting that it may be unique in nitrogen metabolism. In addition, through comparative transcriptomic and proteomic studies on autotrophic and heterotrophic Cp0710, we generated a global view regarding the changes in metabolic pathways under the two growth conditions. We observed that under heterotrophic conditions, enzymes involved in photosynthesis and CO_2_ fixation are almost completely degraded, either as mRNAs or as proteins. Meanwhile, the cells are capable of not only quickly absorbing glucose but also accelerating glucose catabolism through the upregulation of glycolysis and the TCA cycle, which can presumably provide enough energy for both rapid cell growth and enhanced lipid synthesis. Moreover, the rapid synthesis of pyruvate, the upregulation of most enzymes involved in fatty acid synthesis, and the downregulation of enzymes involved in fatty acid degradation favor the accumulation of fatty acids within the cell. This suggests that Cp0710 encodes a fine-tuned pathway for the accumulation of large amounts of lipid under heterotrophic growth conditions.

## Methods

### Strain and culture conditions

The microalga *C. protothecoides* strain 0710 was described previously [22]. The medium and culture conditions were the same as described in our previous study [6]. For autotrophic cultivation, the concentration of glycine used was 5 g/L. For heterotrophic cultivation, glucose was added at a concentration of 10 g/L and glycine was reduced to 0.1 g/L.

### Genome sequencing and assembly

Harvested cells were ground in liquid nitrogen and the total DNA was isolated using the EZgene TM CP Plant Miniprep Kit (GD2621) following the manufacturer’s protocol. A 300-bp (short-insert) and 2-kb (mate-pair) DNA library were prepared according to standard Illumina DNA preparation protocols and sequenced on an Illumina HiSeq 2000 platform. A 400–800-bp DNA library was prepared according to the standard Roche 454 DNA preparation protocol and sequenced on the Roche 454 GS FLX platform. Sequencing data from HiSeq 2000 and Roche 454 GS FLX platforms were assembled into contigs using SOAP *de novo* 2.01 [37] and Newbler V2.6, respectively [38], and further combined with Amos 3.1.0 [39]. Paired-end information from all reads was used for scaffold building from contigs. Local assembly was applied to fill intra-scaffold gaps with reads from a read pair, in which one read aligned within a contig uniquely and the other located within the gap. Assembly and annotation data have been uploaded with the version number APJO01000000 on DDBJ/EMBL/GenBank (accession APJO00000000). Sequence reads, including both genome and transcriptomes, have been deposited into the NCBI sequence read archive (accessions SRA115225).

### Genome annotation

Gene annotation of the Cp0710 genome was accomplished through both a *de novo*-based approach (Augustus v2.5.5 [40], SNAP [41], and Glimmer HMM v3.02 [42]) and a homolog-based approach. The generated gene sets were further combined into a single consensus gene set by applying GLEAN v1.1 [43].

Transcriptome data was also used during the gene annotation of the Cp0710 genome. RNA-seq reads were aligned and assembled into transcripts by Tophat v2.0.4 [44] and Cufflinks v2.0.2 [45], respectively.

Gene sets from genome sequencing data and transcriptome sequencing data were further compared to generate one complete set of genes. For those transcriptome-derived genes with complete ORF structure, if a transcriptome-derived gene covered several genome-derived genes, then these genome-derived genes were replaced by the transcriptome-derived gene. Those transcriptome-derived genes without complete ORF structure were further used to help identify the start and stop site of incomplete genes annotated through the homology-based approach.

Functions were assigned to genes based on the best alignments using Blastp (E-value < = 1e-05) against the Swiss-Prot (release 15.10) [46] and KEGG (Release 60.0) [47] protein databases.

Applying InterProScan v4.7 [39], motifs and domains were annotated by searching against publicly available databases, including ProDom, PRINTS, Pfam, SMART, and PROSITE [48] *.* GO (Gene Ontology) descriptions for the individual genes were also obtained from the corresponding information from InterPro.

The *de novo* repetitive sequences, including simple repeats, satellites, LINEs, SINEs, and LTR retrotransposons, were identified using LTR_FINDER v1.05 [49], PILER v1.0 [50], RepeatModeler v1.0.5 (http://www.repeatmasker.org/RepeatModeler.html), and RepeatScout v1.0.5 [51, 52]. Known repeats were identified with RepeatMasker v3.2.9 (http://repeatmasker.org) against the Repbase v17.06 database [53]. Additionally, repeat-related proteins were identified using RepeatProteinMask against the protein database in Repbase. The results of the two approaches were combined.

### Gene family and phylogenetic tree reconstruction

Gene family identification was performed using TreeFam [54]. BLASTP was used to align all protein sequences against the protein dataset of all species with cut-off E-values of 1e-7. For each gene pair with more than 0.33 of the region aligned to both genes, conjoined fragmental alignments were performed using Solar (Sorting Out Local Alignment Results) (a program in TreeFam). The hierarchical clustering algorithm was applied for gene clustering, with thresholds set to a minimum edge weight of ≥10 and a minimum edge density of ≥0.34.

The protein sequences from 300 single-copy gene families with edge weight >30 and edge density equal to 1, which were conserved among Cp0710, *C. variabilis* NC64A, *C. subellipsoidea* C-169, *C. reinhardtii*, *V. carteri*, *M. pusilla* CCMP1545, and *O. tauri*, were extracted and aligned using MUSCLE v3.8.31 [55]. The multiple alignments were then concatenated into one super sequence for each species. The maximum likelihood tree was constructed with protein sequences using PHYML v3.0 [56] under the WAG with gamma model. Bootstrap values were taken to assess branch reliability.

### Gene comparison and synteny analysis

The orthologues of Cp0710, *C. variabilis* NC64A, and *C. subellipsoidea* C-169 were identified using the gene family identification method described above. The orthologues were classified as core and pairwise orthologues. In addition, the core orthologues was divided into 1:1:1 orthologues and n:n:n orthologues. The 1:1:1 orthologues were defined as single-copy genes presenting in all three species and the n:n:n orthologues represented putative multiple gene duplications in at least one of the species. On the other hand, pairwise orthologues were assigned when orthology was not detectable in the third species. Some genes lacking clear orthology, but with some similarity in the other species were designated as “homologous” (E-value cut-off of 10^-5^ using BlastP).

The synteny blocks between Cp0710 and *C. variabilis* NC64A were identified according to the method described previously [57]. The whole-genome alignments were obtained using the program lastz (Local Alignment Search Tool, blastZ-like, version 1.02.00) in a HOXD55 substitution penalty matrix, and then the aligned gapless segments with the same order and orientation in both species were ligated into “chains” by AXTCHAIN. Subsequently, only a single best alignment for every region of the *C. variabilis* NC64A genome was selected by CHAINNET.

### Transcriptome sequencing and analysis

Total RNAs were isolated from either autotrophic or heterotrophic cells using Trizol Reagent (Invitrogen Inc.), followed by poly (A) mRNA purification with Dynabeads Oligo (dT) (Invitrogen Inc.). The 200-bp cDNA libraries were prepared according to the manufacturer’s instructions (Illumina Inc.). The cDNA libraries derived from enriched mRNA were paired-end sequenced using the Illumina HiSeq™ 2000.

The unigene expression level was determined using RPKM [58], and statistical differences in expression of unigenes was determined using SAGE [59]. To rigorously identify differentially expressed genes with multiple pairwise comparisons, false discovery rate (FDR) correction was applied to constrain errors [60]. Finally, unigenes with an FDR < = 0.001 and an RPKM change of > =2-fold were marked as significantly differentially expressed genes (DEGs), between the two samples.

KEGG [47] pathway enrichment and GO [61] functional enrichment were then carried out for all DEGs. All upregulated or downregulated genes were initially mapped to KEGG or GO terms in the database (KEGG, release 58; GO, release 2012-07-01) using Blastp, then proteins with E-value ≤ 10^-5^ and coverage ≥ 50% were identified. The gene numbers for each term were calculated. Those significantly enriched in GO and KEGG terms were identified by comparison to the genome background using an ultra geometric test with Bonferroni correction, with statistical significance set at P = 0.05. For GO enrichment, to remove redundancy, if the GOs enrich at different levels with parent–child relationship and have the same gene list, the lowest level is chosen.

### Protein preparation and identification

Cells were collected by centrifugation and resuspended in protein extraction buffer (HEPES-40mM, KCl-10mM, MgCl_2_-10mM, EGTA-5mM, CaCl_2_-10mM, PVPP- 0.1%, PMSF-1mM, and DTT-1mM). Then, the cells were disrupted with a mini-BeadBeater, and the mixture was centrifuged at 15,000 × *g* for 10 min, and the supernatant was collected. One volume of SDS/sucrose buffer (sucrose 60%, SDS 4%, and 0.1M Tris–HCl, pH 6.8) was added to the supernatant and blended followed by the addition of one volume of Tris-saturated phenol and vortexed for 10 minutes. After centrifugation at 20,000 × *g* for 10 minutes, the protein-containing layer was collected, and five volumes of a 0.1 M ice cold ammonium acetate–methanol mix was added. The mixture was then incubated at -20°C overnight for protein precipitation. After centrifugation (20,000 × *g* for 10 min), the pellet was washed three times with 0.1 M ammonium acetate–methanol and twice with acetone. Finally, the protein was lyophilized and dissolved in sample buffer for SDS-PAGE.

Protein identification was performed as in a previous study [62]. In brief, 100 μg of protein was separated by one dimensional SDS-PAGE. Each lane was cut into 40 slices, and gel digestion was performed for each slice. The digested samples (20 μl) were analyzed by LC-MS/MS with an LTQ Orbitrap Velosmass spectrometer (Thermo Scientific™). Then, 4 μl of each sample was loaded and gradient eluted with a flow rate of 120 nL/min. For the first 2 minutes, the acetonitrile (ACN) content in the wash buffer was 8%, and in the subsequent 43 minutes the ACN content increased to 55%, in the last 15 minutes the ACN content was 95%. The MS analysis was performed in the Orbitrap at 60,000 full width at half-maximum (FWHM) resolutions, and the 20 most abundant ions were selected for the MS/MS analysis performed in the LTQ.

To identify the proteins, the data from mass spectra were searched against the protein database of Cp0710 using SEQUEST of Thermo Proteome Discoverer 1.2.0.208. The tolerance for peptide identification was 20 ppm in MS and 0.8 Da in MS/MS, and the considered modifications included carbamidomethylation and oxidation. Two missed cleavages were permitted. For quantification, only proteins with PSM ≥ 5 were included in the calculation. The PSM and the total peak area of all peptides for a particular protein were divided by the sum of all PSMs or peak areas of that spectrum, respectively, i.e. normalization to total detected peptides within each sample (central tendency normalization [63]) (Sheet Pro with PSM > =5, Column J to Q in Additional file 2 Table S10). Then, the protein data were compared between the two samples (Sheet Pro with PSM > =5, Colum R and S in Additional file 2 Table S10) to determine the relative change in expression level. This experiment was done twice and only proteins with PSM and area changes ≥ 1.5-fold in both experiments were regarded as significant hits. The raw data from the protein identification is presented in Sheet protein identification in Additional file 2 Table S10. All proteomic data have been submitted to the peptide atlas database (Identifier: PASS00452, http://www.peptideatlas.org/PASS/PASS00452).

## Electronic supplementary material

Additional file 1: **Supplementary information: A: Supplementary Figures S1-S11 B: Supplementary Tables S1-S9.** (DOCX 11 MB)

Additional file 2: Table S10: The proteins differentially expressed in the main pathways of autotrophic and heterotrophic *C. protothecoides*. (XLSX 2 MB)

Additional file 3: Table S11: Functional annotation and pathway analysis of the 2,120 differentially expressed genes using KEGG annotation. (XLSX 16 KB)

Additional file 4: Table S12: Functional annotation and pathway analysis of the 2,120 differentially expressed genes using GO annotation. (XLSX 21 KB)
