# Supplementary material for: Oil accumulation mechanisms of the oleaginous microalga Chlorella protothecoides revealed through its genome, transcriptomes, and proteomes
Source: BMC Genomics. 2014 Jul 10;15(1):582. doi: 10.1186/1471-2164-15-582 (PMC4111847; doi:10.1186/1471-2164-15-582)
Supplement: Supplementary file 1 — Additional file 1: Supplementary information: A: Supplementary Figures S1-S11 B: Supplementary Tables S1-S9. (DOCX 11 MB) [file 12864_2013_6276_MOESM1_ESM.docx]

**Supplemental Materials**

**Oil accumulation mechanisms of the oleaginous microalga *Chlorella protothecoides* revealed through its genome, transcriptomes and proteomes**

Chunfang Gao^1,#,$^ Email: [n1cfgao@sina.com](mailto:n1cfgao@sina.com)

Yun Wang^2,#^ Email: [wangyun@genomics.cn](mailto:wangyun@genomics.cn)

Yue Shen^2,#^ Email: [shenyue@genomics.cn](mailto:shenyue@genomics.cn)

Dong Yan^1^ Email: [severedong588@163.com](mailto:severedong588@163.com)

Xi He^1^ Email: [337979924@qq.com](mailto:337979924@qq.com)

Junbiao Dai^1,*^ Email: jbdai@tsinghua.edu.cn

Qingyu Wu^1,*^ Email: [qingyu@tsinghua.edu.cn](mailto:qingyu@tsinghua.edu.cn)

^1^MOE Key Laboratory of Bioinformatics, School of Life Sciences, Tsinghua University, Beijing 100084, China

^2^BGI-Shenzhen, Shenzhen 518083, China

^#^ These authors contributed equally to this work.

^$^ Current address: Department of Criminal Science and Technology, People's Public Security University of China, Beijing 100038, China

***Corresponding authors:**

Dr. Junbiao Dai, Center for Epigenetics and Chromatin, School of Life Sciences, Tsinghua University, Beijing 100084, China; Phone: +86-10-62796190; Fax: +86-10-62796190; E-mail: [jbdai@tsinghua.edu.cn](mailto:jbdai@tsinghua.edu.cn).

Dr. Qingyu Wu, School of Life Sciences, Tsinghua University, Beijing 100084, China; Phone: +86-10-62781825; Fax: +86-10-62781825; E-mail: [qingyu@mail.tsinghua.edu.cn](mailto:qingyu@mail.tsinghua.edu.cn)

**Supplemental Figure 1**: Pulsed field gel electrophoresis of *C. protothecoides* chromosomes.

Electrophoresis conditions were as follows: 1% agarose gel in 0.5× TBE buffer with pulse ramped from 47 sec to 188 sec for 18 h at 5 V/cm.

**Supplemental Figure 2**: 17 kmer estimation of genome size.

The genome size of *C. protothecoides* sp. 0710 was estimated to be 27.6 Mb based on reads from short insert size library.

**Supplemental Figure 3**: Comparison of genes among *C. protothecoides* sp 0710, *C. variabilis* NC64A and *C. subellipsoidea* C-169. Every gene from each genome was annotated and clustered based on GO. Each bar represents the number of genes and different species is color-coded.

**Supplemental Figure 4:** The gene families of four sequenced green algae. *C. protothecoides, C. variabilis* NC64A*, C. subellipsoidea* C-169 and *C. reinhardtii* were compared. The venn diagram showed the shared and unique gene families. Numbers of gene families are indicated in black. CPRO: *C. protothecoides, CVAR: C. variabilis NC64A; CSUB: C. subellipsoidea C-169; CREI: C. reinhardtii.*

**Supplemental Figure 5:** The phylogenetic tree of H^+^/hexose cotransporter homologous in the seven green algae with genome sequenced (*C. protothecoides*, *C. variabilis* NC64, *C. subellipsoidea* C-169, *C. reinhardtii*, *V. carteri*, *M. pusilla* CCMP1545 and *O. tauri*). The phylogenetic tree was generated by Neighbor-Joining method in MEGA 4, and the bootstrap was set as 1000. All the homologous were categorized into three classes, and two of the branches which contained proteins in all of the seven algae were defined as common proteins. However, the third branch which included the three HUP proteins was only presented in three *Chlorella* species and the other related alga *C. subellipsoidea* C-169, and this group was defined as the HUP-like protein. This kind of protein may be restricted to *Chlorella*.

(The Study Accession URL: <http://purl.org/phylo/treebase/phylows/study/TB2:S15529>)

**Supplemental Figure 6:** Sequence comparison of HUP1 and the three HUP-like proteins (Cpr004256.1, Cpr001753.1 and Cpr003452.1) in *C. protothecoides*.

The 12 transmembrane helixes in Cpr004256.1, Cpr001753.1 and Cpr003452.1 were predicted according to the structure of HUP1. The 6 conserved amino acids responsible for hexose recognition were labeled in red.

**Supplemental Figure 7:** The main process in the proteomic study of autotrophic and heterotrophic *C. protothecoides*.

After one-dimension protein separation, each lane was cut into 23 stripes, and every of which was digested in gel by trypsin, separately. Then, every sample was analyzed by LC/MS/MS (LTQ Orbitrap Velos). In protein identification, the 23 results of each lane were combined and searched against the protein database of *C. protothecoides* with the program of SEQUEST. As a result, 1931 proteins were identified in autotrophic and heterotrophic cells. PSM and area in protein identification were used for the different expression analysis. In heterotrophic cells, 205 proteins are up-regulated (both PSM and area are increased ≥ 1.5 fold) and 293 proteins are down-regulated (both PSM and area are decreased ≥ 1.5 fold)

**Supplemental Figure 8:** Comparison of transcriptome and proteome in autotrophic and heterotrophic cell.

ARNA and HRNA: transcriptomic gene expression in autotrophic and heterotrophic cell respectively (coverage >=50% or rpkm>=10); APROT and HPROT: proteomic gene expression in autotrophic and heterotrophic cell respectively (PSM>=5).

**Supplemental Figure 9:** The functional classification of the genes differently expressed in comparative transcriptomic analysis of autotrophic and heterotrophic *C. protothecoides*.

After mRNA sequencing, the expression levels of them in autotrophic and heterotrophic conditions were compared, and the genes with log2 (fold-change) >1 and P < 0.01 were defined as significantly changed. As a result, in heterotrophic cells 984 genes were up-regulated and 1136 genes were down-regulated. All of the genes were classified to 9 clusters according to KEGG metabolism pathway annotation.

**Supplemental Figure 10.** The elimination of chloroplast after transition from autotrophic to heterotrophic growth.

(**A**) Visualization of chloroplast or lipid body by confocal microscopy in autotrophic (upper) and heterotrophic (lower) cells. Red, Nile Red fluorescence used to show the lipid body. Green, chlorophyll autofluorescence pseudocolored in green, indicating the presence of chloroplast. (**B**) The ultrastructure of *C. protothecoides* cultivated in autotrophic and heterotrophic condition using transmission electron microscopy (TEM). The autotrophic cell (left) contained a cuplike chloroplast (CP). The heterotrophic cell (right) contained a big lipid body (LB).


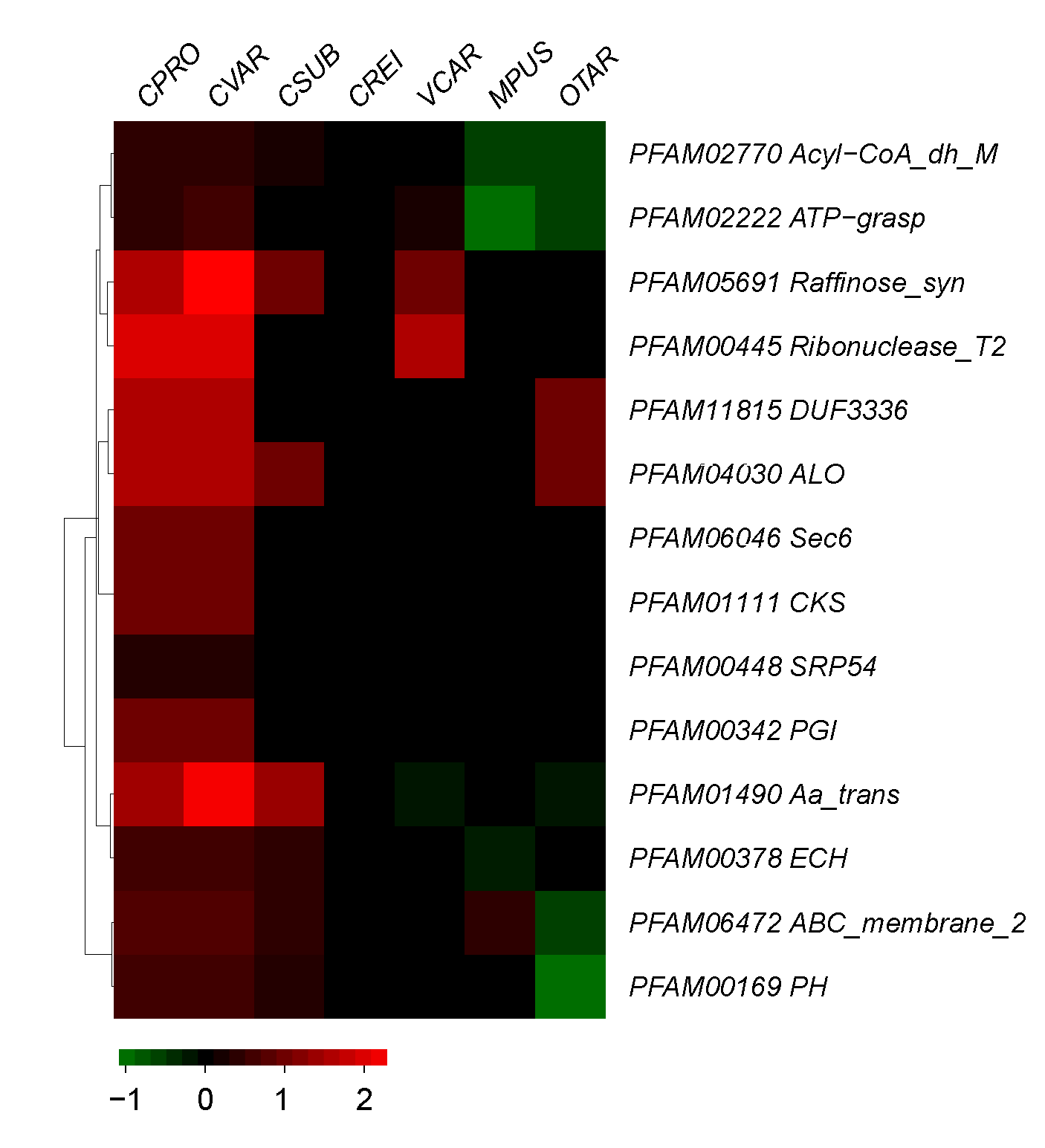


**Supplemental Figure 11:** The heat map of PFAM domain with biased distribution in *C. protothecoides*.

The number in *C. reinhardtii* were used as the reference and indicated in black. Red and green indicated the higher or lower numbers of PFAM in different algae. CPRO: *C. protothecoides, CVAR: C. variabilis NC64A; CSUB: C. subellipsoidea C-169; CREI: C. reinhardtii. VCAR: V. carteri; MPUS: M. pusilla CCMP1545; OTAR: O. tauri.*

Supplemental Table 1: *Chlorella protothecoides* sp. 0710 17 kmer statistics

| Species | K | K_num | K_depth | Genome_size | X |
| --- | --- | --- | --- | --- | --- |
| *C. protothecoides* | 17 | 414,205,607 | 15 | 27,613,707 | 16.70 |

Supplemental Table 2: Statistics of the completeness of the genome based on 248 CEGs

|  | #Prots | %Completeness | - | #Total | Average | %Ortho |
| --- | --- | --- | --- | --- | --- | --- |
| Complete | 225 | 90.73 | - | 274 | 1.22 | 19.56 |
| Group 1 | 56 | 84.85 | - | 62 | 1.11 | 8.93 |
| Group 2 | 51 | 91.07 | - | 64 | 1.25 | 21.57 |
| Group 3 | 58 | 95.08 | - | 75 | 1.29 | 25.86 |
| Group 4 | 60 | 92.31 | - | 73 | 1.22 | 21.67 |
|  |  |  |  |  |  |  |
| Partial | 236 | 95.16 | - | 300 | 1.27 | 23.31 |
| Group 1 | 60 | 90.91 | - | 71 | 1.18 | 15 |
| Group 2 | 53 | 94.64 | - | 73 | 1.38 | 28.3 |
| Group 3 | 59 | 96.72 | - | 77 | 1.31 | 27.12 |
| Group 4 | 64 | 98.46 | - | 79 | 1.23 | 23.44 |

“Prots” indicated number of 248 ultra-conserved CEGs present in genome; “% Completeness” indicates percentage of 248 ultra-conserved CEGs present; “Total” indicates total number of CEGs present including putative orthologs; “Average” indicates average number of orthologs per CEG; “%Ortho” indicates percentage of detected CEGS that have more than 1 ortholog.

| Type | Repeat Size ^a^ | % of genome |
| --- | --- | --- |
| Trf | 431,366 | 1.8817 |
| Repeatmasker | 544,464 | 2.3750 |
| Proteinmask | 564,023 | 2.4603 |
| De novo | 1,028,311 | 4.4856 |
| Total | 1,397,655 | 6.0967 |

Supplemental Table 3: Repeats in the genome of Cp0710 with combined approaches.

^a^ The overlaps between repeats have been excluded before the calculation .

Supplemental Table 4: Results of repeat prediction without TRF.

| Type | RepeatMasker | | ProteinMasker | | Denovo | | Combined ^a^ | |
| --- | --- | --- | --- | --- | --- | --- | --- | --- |
|  | Length (Bp) | % in genome | Length (Bp) | % in genome | Length (Bp) | % in genome | Length (Bp) | % in genome |
| DNA | 5,342 | 0.0233 | 6,327 | 0.0276 | 84,696 | 0.3695 | 96,207 | 0.4197 |
| LINE | 4,431 | 0.0193 | 96 | 0.0004 | 84,183 | 0.3672 | 88,710 | 0.3870 |
| SINE | 642 | 0.0028 | 0 | 0.0000 | 0 | 0.0000 | 642 | 0.0028 |
| LTR | 15,007 | 0.0655 | 14,494 | 0.0632 | 41,173 | 0.1796 | 66,527 | 0.2902 |
| Satellite | 851 | 0.0037 | 449 | 0.0020 | 2,919 | 0.0127 | 2,921 | 0.0127 |
| Simple repeat | 140,139 | 0.6113 | 139,387 | 0.6080 | 153,278 | 0.6686 | 197,878 | 0.8632 |
| Low complexity | 379,791 | 1.6567 | 330,534 | 1.4418 | 373,521 | 1.6293 | 399,048 | 1.7407 |
| Tandem Repeat | 0 | 0 | 73,073 | 0.3188 | 0 | 0.0000 | 73,073 | 0.3188 |
| Unknown^b^ | 210 | 0.0009 | 0 | 0.0000 | 302,440 | 1.3193 | 302,650 | 1.3202 |
| Total | 544,464 | 2.3750 | 564,023 | 2.4603 | 1028,311 | 4.4856 | 1,123,650 | 4.9015 |

^a^ The length of repeats was calculated and overlaps have been excluded before.

^b^ This refers to the repeats that can’t be classified by RepeatMasker.

Supplemental Table 5: General statistics of predicted protein-coding genes

|  | De novo | | | Homolog | | | GLEAN |
| --- | --- | --- | --- | --- | --- | --- | --- |
|  | Augustus | SNAP | GlimmerHMM | CREI | CVAR | CSUB |  |
| Total number of genes | 5473 | 7446 | 20068 | 4302 | 5166 | 4782 | 6247 |
| Average length of mRNA | 2960.31 | 2379.82 | 1068.94 | 1553.34 | 1948.45 | 1714.04 | 2598.03 |
| Average length of cds | 1413.21 | 1137.91 | 937.18 | 888.11 | 1030.37 | 950.78 | 1292.44 |
| Average number of exon | 7.30 | 6.35 | 3.10 | 3.91 | 4.65 | 4.23 | 6.28 |
| Average length of exon | 193.66 | 179.10 | 301.90 | 227.32 | 221.67 | 224.60 | 205.73 |
| Average length of intron | 245.67 | 231.99 | 62.61 | 228.85 | 251.65 | 236.07 | 247.17 |
| Total number of exon | 39939 | 47307 | 62297 | 16807 | 24013 | 20243 | 39245 |
| Total number of intron | 34466 | 39861 | 42229 | 12505 | 18847 | 15461 | 32998 |

CREI: *Chlamydomonas reinhardtii*; CVAR: *Chlorella variabilis* NC64A; CSUB: *Coccomyxa subellipsoidea* C-169.

Supplemental Table 6: Statistics of functional annotation

| Type | Database | Gene | Percentage (%) |
| --- | --- | --- | --- |
| Total gene | | 7039 | 100 |
| Annotated | TrEMBL | 5725 | 81.33 |
|  | Swissprot | 4537 | 64.46 |
|  | KEGG | 3910 | 55.55 |
|  | InterPro | 4599 | 65.34 |
|  | GO | 3559 | 50.56 |
| All annotated | | 5831 | 82.87 |

Supplemental Table 7: Proteins involved in nitrogen transport and assimilation in green algae ^a^.

| Reference proteins ^b^ | MPUS | OTAR | VCAR | CSUB | CVAR | CPRO |
| --- | --- | --- | --- | --- | --- | --- |
| **Transporter** |  |  |  |  |  |  |
| XP_001694496.1  nitrate transporter | XP_003057942.1 | XP_003081529.1 | EFJ43737.1 | EIE23748.1 | EFN52690.1 |  |
| XP_001694067.1  nitrite transporter | XP_003058319.1 | XP_003081525.1 | EFJ43209.1 | EIE18297.1 | EFN58263.1 | Cpr000340.1 |
| XP_001701575.1  ammonium transporter | XP_003063809.1 | XP_003084401.1 | EFJ40601.1 | EIE23179.1 | EFN53204.1 | Cpr001664.1 |
| XP_001691580.1  urea active transporter |  | XP_003083319.1 | EFJ41618.1 | EIE20547.1 | EFN55634.1 |  |
| XP_001694885.1  amino acid transporter |  | XP_003074291.1 | EFJ48238.1 | EIE22399.1 | EFN60084.1 | Cpr002375.1 |
| **Assimilation** |  |  |  |  |  |  |
| XP_001696697.1  nitrate reductase | XP_003058323.1 | XP_003081526.1 | EFJ43675.1 | EIE21865.1 | EFN52691.1 | Cpr000877.1 |
| XP_001696787.1  nitrite reductase | XP_003057941.1 | XP_003081527.1 | EFJ43735.1 | EIE21866.1 | EFN52613.1 | Cpr001933.9 |
| NP_176922.1 ^c^  urease |  | XP_003083318.1 |  |  |  |  |
| NP_173602.1 ^c^  urease accessory protein F |  | XP_003083320.1 |  |  |  |  |
| AAD16984.1 ^c^  urease accessory protein UREG |  | XP_003083317.1 |  |  |  |  |
| NP_850239.1 ^c^  urease accessory protein D |  | XP_003078095.1 |  |  | EFN50428.1 |  |
| XP_001692927.1  glutamine synthetase | XP_003057550.1 | XP_003074553.1 | EFJ51602.1 | EIE23502.1 | EFN56917.1 | Cpr003038.1 |
| XP_001693082.1  glutamate synthase | XP_003057676.1 | XP_003083015.1 | EFJ40691.1 | EIE24001.1 | EFN59782.1 | Cpr004691.1 |
| XP_001702270.1  glutamate dehydrogenase |  |  | EFJ45751.1 | EIE23148.1 | EFN55208.1 | Cpr003571.2 |
| XP_001703658.1  nitrogen regulatory protein PII | XP_003062922.1 |  | EFJ41943.1 |  | EFN50797.1 | Cpr004333.1 |
| **Urea Cycle** |  |  |  |  |  |  |
| XP_001690709.1  carbamoyl phosphate synthase,  large subunit | XP_003058904.1 | XP_003080815.1 | EFJ40705.1 | EIE20839.1 | EFN52062.1 | Cpr002904.1 |
| XP_001690929.1  ornithine carbamoyltransferase | XP_003056510.1 | XP_003074205.1 | EFJ50768.1 | EIE22039.1 | EFN58131.1 | Cpr003449.1 |
| XP_001696749.1  argininosuccinate synthase | XP_003063309.1 | XP_003083741.1 | EFJ48533.1 | EIE18346.1 | EFN55601.1 | Cpr002598.3 |
| CAA34615.1  argininosuccinate lyase | XP_003055265.1 | XP_003082446.1 | EFJ46826.1 | EIE22869.1 | EFN52305.1 | Cpr003750.5 |
| NP_192629.1 ^c^  arginase |  |  |  |  |  |  |

^a^ Only one protein with the highest score in BlastP (E-value<1E-5) were listed.

^b^ Most of the reference proteins used for BlastP were proteins of *C. reinhardtii*.

^c^ The proteins of *Arabidopsis thaliana* were used when they were not found in *C. reinhardtii*. MPUS: *Micromonas pusill*a CCMP1545; OTAR: *Ostreococcus tauri*; VCAR: *Volvox carteri*; CSUB: *Coccomyxa subellipsoidea* C-169; CVAR: *Chlorella variabilis* NC64A; CPRO: *Chlorella protothecoides* sp. 0710.

Supplemental Table 8: H^+^/hexose cotransporters in *Chlorella kessleri* and their homologs in green algae (BlastP, E-value<1E-5, Alignment length>30%)

| Green algae | Homologs |
| --- | --- |
| CKES | P15686.2 Q39524.1 Q39525.1 |
| CPRO | Cpr004256.1 Cpr001753.1 Cpr003452.1 Cpr002964.1 Cpr005023.1 Cpr003677.1 Cpr003720.1 Cpr003252.1 Cpr003700.3 |
| CVAR | EFN53774.1 EFN53666.1 EFN50678.1 EFN59533.1 EFN55620.1 EFN60010.1 EFN50549.1 EFN54539.1 EFN58991.1 EFN50679.1 EFN53468.1 EFN59043.1 EFN52027.1 |
| CSUB | EIE21809.1 EIE25646.1 EIE22313.1 EIE22314.1 EIE19027.1 EIE27221.1 EIE20603.1 EIE22323.1 EIE25022.1 EIE21778.1 EIE25408.1 EIE22371.1 EIE26813.1 EIE26164.1 EIE25526.1 EIE20660.1 EIE21964.1 EIE20094.1 EIE20926.1 |
| CREI | XP_001693177.1 XP_001701103.1 |
| VCAR | EFJ48518.1 EFJ42942.1 |
| MPUS | XP_003063909.1 XP_003062688.1 XP_003054757.1 XP_003062182.1 XP_003059968.1 XP_003059519.1 |
| OTAR | XP_003082978.1 XP_003078139.1 XP_003077948.1 XP_003080102.1 |

CKES: *Chlorella Kessler*; CPRO: *Chlorella protothecoides* sp. 0710; CVAR: *Chlorella variabilis* NC64A; CSUB: *Coccomyxa subellipsoidea* C-169; CREI: *Chlamydomonas reinhardtii*; VCAR: *Volvox carteri*; MPUS: *Micromonas pusilla* CCMP1545; OTAR: *Ostreococcus tauri*.

Supplemental Table 9: Transcriptome sequencing data statistics.

| Culture condition | Total raw reads (M) | Total raw base (G) | Total clean reads (M) | Total clean base (G) | Reads map to genome (M) | Mapping ratio (%) |
| --- | --- | --- | --- | --- | --- | --- |
| Autotrophy | 43.7 | 3.93 | 40.2 | 3.62 | 32.4 | 80.7 |
| Heterotrophy | 40.9 | 3.68 | 37.9 | 3.41 | 30.8 | 81.2 |

**Supplemental Methods**

**Pulse Field Gel Electrophoresis**

The Pulse field gel electrophoresis (PFGE) was carried out according to Blanc et al.^1^, with slight modifications. The algal cells were harvested from 4 day old cultures by centrifugation at 5,000×*g* for 5 minutes. Approximately 0.25 ml cell pellets were re-suspended in 200 μl deionized water, mixed with 2% low melting point agarose in 100 mM EDTA at 42°C, poured into plug molds (Bio-Rad), and placed at 4°C for about 20-30 minutes to harden the agarose. Once solidified, all the plugs were incubatd with solution V (500mM EDTA, 10mM Tris, pH7.5) overnight at 37°C. On the next day 400 μl of 5 mg/ml proteinase K in 500 mM EDTA (pH7.5) was added to the tube and gently inverted a few times to homogenize the solution. They were then incubated overnight and washed with deionized water. Subsequent washes were done using 1× TE in the cold room overnight. Electrophoresis was performed under following condition: 1% agarose gel in 0.5× TBE buffer with pulse ramped from 47 sec to 188 sec for 18 h at 5 V/cm. *Saccharomyces cerevisia*e chromosomes were used as the PFGE markers (225 to 1900kb).

**Supplemental Refereneces**

1. Blanc, G. et al. The genome of the polar eukaryotic microalga Coccomyxa subellipsoidea reveals traits of cold adaptation. *Genome biology* **13**, R39 (2012).
